# Syndrome de larva migrans cutanée sur pied malformé (à propos d'un cas)

**DOI:** 10.11604/pamj.2016.23.50.8696

**Published:** 2016-02-19

**Authors:** Imane Benbella, Hanane Khalki, Khalid Lahmadi, Sara Kouara, Abderrahim Abbadi, Mohammed Er-rami

**Affiliations:** 1 Service de Parasitologie et de Mycologie, Hôpital Militaire de Moulay Ismail, Meknès, Maroc

**Keywords:** Larva migrans, nourrisson, cutané, malformation, Larva migrans, infant, cutaneous, malformation

## Abstract

Le syndrome de larva migrans cutanée est une dermite sous cutanée causée par des larves d'ankylostomes d'animaux en impasse parasitaire chez l'homme. L'infestation transcutanée est favorisée par le contact avec le sol contaminé par les larves du parasite. Nous rapportons le cas d'un nourrisson de 15 mois, originaire de Guinée-Bissau, atteint d'un syndrome de larva migrans cutanée sur un pied malformé. Cette malformation sous forme d'une syndactylie associée à une tuméfaction du pied, était à l'origine d'un retard d'acquisition de la station debout. De même, on a rapporté une notion de pieds nus, vue la difficulté de chausser le pied malformé du patient. Tous ces facteurs auraient contribués à favoriser l'infestation du malade par les larves du nématode.

## Abstract (en)

Cutaneous larva migrans syndrome is a subcutaneous dermatitis caused by hookworms’ larvae, originating from animals in parasitic impasse in humans. Transcutaneous infestation is favored by contact with contaminated soil. We report the case of a 15-month-old child, native of Guinea - Bissau, suffering from cutaneous larva migrans syndrome on a malformed foot. This malformation in the form of a syndactyly, associated with a tumefaction of the foot cause a delay in the standing position. Besides, the fact that the child never wears shoes because of the sick foot is another factor contributing to the patient's infestation by the larvae of the nematode.

## Introduction

Le syndrome de larva migrans cutanée est une dermite rampante, causée par l'infestation accidentelle et la migration d'une larve de nématode en impasse parasitaire chez l'homme dont la pénétration larvaire se fait par voie transcutanée [1]. Cette affection, est souvent retrouvée en zone tropicale et subtropicale, où le climat chaud et humide favorise la viabilité des larves infestantes. La marche pieds nus sur des sols boueux, ensablés ou des plages, présente un risque élevé de contamination [2]. La surface cutanée serait plus importante chez les nourrissons en âge de reptation ou ayant un retard de marche. Nous rapportons le cas d'un nourrisson âgé de quinze mois, originaire de Guinée-Bissau, atteint d'un syndrome de larva migrans cutanée sur un pied malformé et n'ayant pas encore acquis la station debout.

## Patient et observation

**Figure 1:**
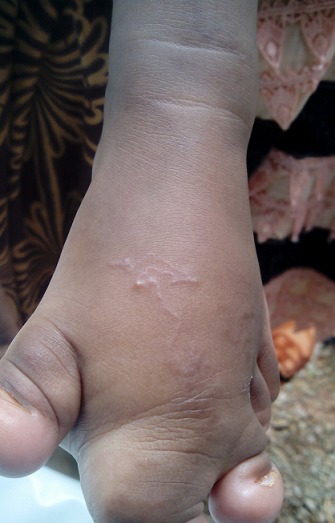
Trajet sinueux et serpigineux correspondant au trajet de migration de la larve au niveau du pied gauche

**Figure 2:**
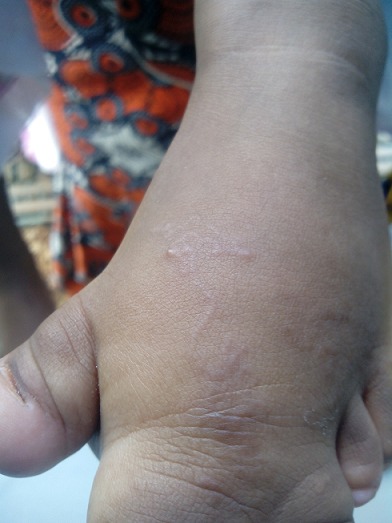
Début d'atténuation de la lésion après 24 heures de traitement

Il s'agit d'un nourrisson âgé de quinze mois vivant à Bissau (capitale de Guinée-Bissau) qui a été amené en consultation pour une lésion prurigineuse au niveau du pied gauche évoluant depuis une semaine. L'anamnèse rapporte une exposition cutanée prolongée aux sols, vu le retard d'acquisition de la station debout, et la difficulté de chausser le patient. En effet, l'examen clinique a trouvé un patient qui présente des difficultés à maintenir la position debout. L'examen du pied a révélé un pied gauche malformé, avec une tuméfaction et une syndactylie des 2^e^ et 3^e^ orteils. On a noté la présence d'une lésion au trajet sinueux, au sein duquel on a observé un cordon serpigineux (Figure 1). Le tout évoluait dans un contexte de conservation de l’état général et d'apyrexie. Le bilan biologique était sans particularité. Le patient a été mis sous albendazole à la dose de 200mg/j pendant trois jours. Une atténuation de la lésion a été observée après 24 heures de traitement (Figure 2).

## Discussion

Le syndrome de larva migrans cutanée, également appelé larbish, ou dermite ankylostomienne, fut décris pour la première fois en 1874 [3]. Il correspond à la pénétration active en transcutanée, chez l'Homme, de larves d'ankylostomes se trouvant dans des sols humides et chauds souillés par les défections des animaux notamment les chiens, les chats, et les autres mammifères [1, 2, 4, 5]. Cette dermatite, est causée essentiellement par Ancylostoma braziliense, Ancylostoma caninum, Ancylostoma ceylonium, Uncinaria steoncephala, Bubostomum phlebotomus [3]. Des cas de syndrome de larva migrans cutanée chez les nourrissons en âge de reptation ont été rapportés [3]. Notre patient, quant à lui, présentait un retard d'acquisition de station debout, du fait de sa malformation podale, ce qui aurait limité ses déplacements, prolongeant ainsi le contact avec le sol contaminé. La plupart de ces larves, naturellement inféodées aux animaux, sont incapables de poursuivre leur cycle chez l'Homme et meurent entre les deux à huit semaines faisant suite à l'infestation [6]. Les larves d'ankylostomes pénètrent la peau à travers les follicules pileux et les pores des glandes sudorales, mais elles peuvent également pénétrer la surface cutanée en l'absence de toute effraction [6]. La migration larvaire se fait le plus souvent la nuit, à travers l’épiderme et le derme superficiel [6]. Alors que la larve creuse à travers la peau, une réaction inflammatoire est observée à cause de l'antigenicité du parasite et de ses sécrétions protéolytiques (notamment les hyaluronidases), expliquant ainsi les caractéristiques inflammatoires, prurigineuses et serpigineuses de la lésion [6]. En effet, le signe initial et pathognomonique est décrit, comme une lésion linéaire ou serpigineuse érythémateuse, voire papulovésiculeuse, légèrement surélevée, qui migre en amont selon un mouvement irrégulier et selon un rythme de 2-3mm/j [3, 7]. Cette lésion apparaît le plus souvent dans les cinq premiers jours suivant la pénétration larvaire de la peau, toutefois, la période d'incubation peut être plus longue chez les voyageurs [7]. Dans la plupart des cas, un prurit intense, souvent décrit comme inconfortable, est retrouvé [7]. Par ailleurs, une douleur peut également être présente [7]. Une aggravation des lésions, par surinfection bactérienne peut survenir, elle est le plus souvent causée par Staphylococcus aureus et Strepctococcus spp, qui viennent se greffer sur les lésions de grattage [7]. Dans des formes plus graves, les patients peuvent développer des folliculites, impétigo, des lésions vésiculo-bulleuses, ou même un syndrome de Loëffer avec une infiltration pulmonaire [7]. Le diagnostic repose sur le tableau clinique et l'anamnèse rapportant à un séjour en zone endémique [4] et/ou un contact prolongé avec du sable, ou des sols humides. D'autres outils diagnostiques peuvent être utiles. Ainsi la microscopie à épiluminescence, permet de façon non invasive de détecter la larve, et donc de confirmer le diagnostic [3]. La biopsie cutanée, la microscopie confocale par reflectance, et la tomographie à cohérence optique, même si leur valeur reste limitée, peuvent également être contributifs au diagnostic [3].

Sur le plan biologique, des anomalies sont rarement rencontrées, mise à part quelques cas d'hyperéosinophilie [5]. Chez notre patient le diagnostic fut posé à la seule base du tableau clinique pathognomonique et du contexte endémique de la région. Le syndrome de larva migrans cutanée est facilement traité [5]. Le traitement de choix étant l'ivermectine, par voie orale, à dose unique de 200mg/kg. Cette dernière permet la destruction efficace des larves migrantes [5]. Dans le cas, où un premier traitement échoue, une seconde dose assure le plus souvent une guérison de manière efficace et définitive [5]. Toutefois, dans les pays ne disposant pas d'ivermectine, la prescription de doses répétées d'albendazole, constitue une bonne alternative thérapeutique. La dose recommandée étant de 400mg/j pendant en moyenne trois à sept jours, avec une bonne réponse dans 92% à 100% des cas [4, 5]. Ce fut le cas pour notre patient qui a présenté une amélioration de ces symptômes dans les 24 heures ayant suivi l'administration de la première dose d'albendazole (200mg/j). Afin de réduire le risque d'infestation par Larva migrans cutanée, des mesures préventives devraient être prises dont la principale est d’éviter le contact avec les sols susceptibles d’être contaminés. Ainsi, le port de chaussures et de sandales est préconisé [8]. Toutefois, de nombreuses personnes, semblent penser, à tort, que l'addition de chaussettes assurerait une protection optimale. En effet, il semblerait que le port de chaussettes en zone sablées ne préserverait pas de la pénétration du sable bien au contraire il prédispose à l'infestation parasitaire [7]. En effet, l'accumulation du sable au niveau des chaussettes, contribuerait à un contact prolongé avec le parasite et par conséquent augmenterait les chances de sa pénétration [3, 7]. D'autres moyens de prévention, peuvent également être mis en place, notamment: l'interdiction des chiens et des chats au niveau des plages et aires de jeux, et leur vermification régulière, par des antihelminthiques, même si cette dernière mesure s'avère d'application relativement difficile, vue les ressources qu'elle implique, d'autant plus qu'il s'agit le plus souvent d'animaux errants [2, 9].

## Conclusion

Le syndrome de larva migrans cutanée est une affection relativement rare et bénigne. La principale cause de contamination humaine reste les animaux domestiques errants. Son diagnostic devrait se baser essentiellement sur l'aspect clinique, qui le plus souvent est pathognomonique. Les nourrissons, surtout avant l'acquisition de la marche, constitueraient une population à risque, vue leur exposition plus fréquente, et plus prolongée au sol.
